# The Risk of Infection from Polychlorinated Biphenyl Exposure in the Harbor Porpoise (*Phocoena phocoena*): A Case–Control Approach

**DOI:** 10.1289/ehp.8222

**Published:** 2006-01-13

**Authors:** Ailsa J. Hall, Kelly Hugunin, Robert Deaville, Robin J. Law, Colin R. Allchin, Paul D. Jepson

**Affiliations:** 1 Sea Mammal Research Unit, Gatty Marine Laboratory, University of St. Andrews, St. Andrews, Fife, United Kingdom; 2 North Carolina State College of Veterinary Medicine, Raleigh, North Carolina, USA; 3 Institute of Zoology, Zoological Society of London, London, United Kingdom; 4 Centre for Environment, Fisheries and Aquaculture Science, Burnham Laboratory, Burnham-on-Crouch, Essex, United Kingdom

**Keywords:** cetaceans, dose response, immunosuppression, PCBs, risk assessment

## Abstract

The objective of this study was to determine whether the risk of mortality from infectious disease in harbor porpoise in U.K. waters increased with high exposure to polychlorinated biphenyls (PCBs), using a case–control study design. This is the first time that data from a long-term marine mammal strandings scheme have been used to estimate any increase in risk. The exposure odds ratio (OR) from a logistic regression model with infectious disease deaths as cases and physical trauma deaths as controls, after controlling for the effect of confounding factors, was 1.048 [95% confidence interval (CI), 1.02–1.07]. To further adjust for the difference in energetic status between cases and controls and account for the negative relationship between PCBs (sum of 25 chlorobiphenyl congeners) and blubber mass, we also “standardized” the blubber PCBs to an optimal blubber mass. This lowered the OR to 1.02 (95% CI, 1.00–1.03). Thus, for each 1 mg/kg increase in blubber PCBs, the average increase in risk of infectious disease mortality was 2%. A doubling of risk occurred at approximately 45 mg/kg lipid. In this study, we have endeavored to avoid selection bias by using controls that died of physical trauma as representative of the exposure prevalence in the population that gave rise to the cases. In addition, we controlled for the effect of variation in energetic status among the cases and controls. However, as with case–control studies in human and veterinary epidemiology, unforeseen misclassification errors may result in biased risk estimates in either direction.

Many studies have reported an association between immune suppression or infectious disease and exposure to polychlorinated biphenyls (PCBs) in laboratory animals, humans, and wildlife (Bernhoft et al. 2000; De Swart et al. 1996; Jepson et al. 2005; Kimbrough 1987; Schwacke et al. 2002; Vos et al. 2000). Because marine mammals are at the top of the food chain and have large lipid stores, they accumulate high levels of PCBs in their blubber (Aguilar et al. 2002; Hansen et al. 2004; Ross et al. 2000). Some small species of seal or porpoise may therefore provide potentially useful mammalian models of PCB-induced toxic effects. Although from a toxicologic perspective it is not possible to carry out controlled exposure experiments on captive marine mammals, population-based epidemiologic studies to determine the risk of specific outcomes after exposure to potential causal agents, such as PCBs, can be employed. By applying epidemiologic principles to studies on wildlife, relative risk estimates for specific exposure–response hypotheses can be obtained. Such studies are widely used in human and veterinary medicine (Khoury and Beaty 1994; Pike et al. 1975; Shapiro 1989) but have received scant attention in relation to wildlife. This is understandable given the logistical difficulties in obtaining long-term disease and exposure data, particularly in marine mammals, which spend most or all of their lives at sea. Follow-up (cohort) studies might be possible for a limited number of species that are subject to long-term longitudinal studies, but case–control studies can be implemented on a wider variety of species and populations. In this study, we demonstrate the use of this approach to estimate the risk of infectious disease death from exposure to PCBs in a marine mammal, using a long-term data set on harbor porpoises in U.K. waters.

Since the late 1980s, the link between persistent organic pollutant (POP) exposure [e.g., PCBs, dichlorodiphenyltrichloroethane (DDT), and chlorinated pesticides] and immunosuppression in marine mammals has received considerable attention (Aguilar and Borrell 1994a; Beckmen et al. 2003; Bernhoft et al. 2000; De Guise et al. 1995; De Swart et al. 1996; Hall et al. 1992, 1997; Jepson et al. 1999, 2005; Kuiken et al. 1994; Lahvis et al. 1995; Lie et al. 2004, 2005; Ross et al. 1995, 1996). A number of mass mortalities from infectious diseases in a variety of species worldwide (Aguilar and Borrell 1994b; Dietz et al. 1989; Geraci 1989; Jensen et al. 2002) heightened interest in the relationship between POPs and immune function. Laboratory studies had already shown these contaminants to be immunosuppressive in a wide variety of species (Dean et al. 1982, 1985). By the 1990s, evidence for a correlation between high blubber levels of POPs, particularly PCBs, in marine mammals and mortality from infectious disease was accumulating. For example, in cetaceans, Aguilar and Borrell (1994b) found that striped dolphins that died during the 1990 morbilli-virus epidemic in the Mediterranean Sea had significantly higher PCBs in their blubber than did animals sampled outside the epidemic (median, 282 mg/kg lipid weight in 1987–1989 and 1991 vs. 778 mg/kg lipid weight in 1990). Similar results had also been seen during the 1987–1988 bottlenose dolphin die-off (Kuehl et al. 1991) and during the 1988 European phocine distemper virus epidemic among harbor seals (Hall et al. 1992).

Further evidence for specific immune function effects of POP exposure in cetaceans came from *in vitro* studies. Lahvis et al. (1995) assessed lymphocyte proliferative responses to mitogen stimulation in five bottlenose dolphins with a range of PCB and DDT blood levels. Significant negative correlations were found, particularly in two animals with blood PCB levels of > 700 ng/g wet weight, whose proliferative responses were < 50% of that of the other three animals. However, age effects were not accounted for in this study. Similarly, beluga whale (*Delphinapterus leucas*) splenocyte proliferative responses were significantly reduced after exposure to mixtures of PCB and DDT congeners at levels 5–25 μg/g wet weight (De Guise et al. 1998). More recently, Lie et al. (2004) found significant negative relationships between high blood levels of PCBs and serum immunoglobulins against a range of pathogens in polar bears (*Ursus maritimus*) and a significant negative relationship between PCB exposure (blood levels of 32–89 ng/g wet weight) and cell-mediated immunity (Lie et al. 2005).

The most comprehensive study of the effects of POPs on immunity in marine mammals involved captive seals (De Swart et al. 1994). Two groups of harbor seals were fed herring from the relatively unpolluted Atlantic and from a highly polluted region of the Baltic Sea. De Swart et al. (1994) and Ross et al. (1996) reported negative effects on natural killer cell activity (important in viral defense) and on T-cell mitogen-induced proliferation. In addition, higher circulating levels of poly-morphonuclear cells were found in animals fed contaminated fish, suggesting an increase in bacterial infections. Ross et al. (1995) also found that contaminant-fed seals were less able to mount a specific immune response to oval-bumin. These authors reported the contaminant concentrations in the blubber of the two groups at the end of the study as toxic equivalents (TEQs), making it difficult to compare the results with other published studies and to infer which groups of contaminants are likely to be responsible for the observed effects. However, reductions in proliferative responses to mitogens have been associated with aryl hydrocarbon receptor (AhR) binding (the binding of contaminants to this soluble cytosolic protein is particularly important in thymus-dependent immune effects) (Safe 1994; Vos et al. 1997), and up to 93% of the AhR-active compounds in the blubber of the seals were PCBs, whereas other potentially immunotoxic compounds such as polychlorinated dibenzo-*p*-dioxins, polychlorinated dibenzofurans, DDT, butyltins, and mercury were found in much smaller proportions, suggesting that immunosuppressive effects were likely to be largely due to PCBs. Kannan et al. (2000) used these results and information published by Boon et al. (1987) and Brouwer et al. (1989) to convert the daily intake of PCBs in the diet to an estimated blood level, thus approximating a lowest observed adverse effect level (LOAEL) for PCB effects on immunity of 16 ng/g wet weight in seal blood. They also approximated a PCB LOAEL (from Lahvis et al. 1995) for dolphins of 26 ng/g wet weight in blood, slightly higher than for the seals.

Small cetaceans in particular may have a lower capacity to metabolize PCBs (Tanabe et al. 1988). Based on their blubber PCB congener patterns, Tanabe et al. (1988) hypothesized that cetaceans do not possess a group of cytochrome P450 enzymes (known as CYP2B isoenzymes) responsible for the oxidation and activation of halogenated aromatic hydrocarbons, suggesting that these species accumulate some of the toxic PCB congeners and are therefore more susceptible to their long-term effects (Boon et al. 2001; Goksøyr et al. 1986). However, more recent studies have identified immunoreactive proteins recognized by heterologous CYP2B antibodies in a variety of cetacean species (Goksøyr 1995; White et al. 1994), including the harbor porpoise (Hummert et al. 1995), and CYP1B-like amino acid sequences from striped dolphin cDNA have also now been reported (Godard et al. 2000). This perhaps now questions the suggestion that small cetaceans in particular are more sensitive to the effects of exposure to POPs.

Attention has continued to focus on the harbor porpoise because high levels of contaminants have been extensively reported in this species (for review, see Aguilar et al. 2002; O’Shea 1999; Reijnders et al. 1999). In addition to the high by-catch rates sustained by some populations (Kock and Benke 1996; Trippel et al. 1999), any additional anthropogenic effects on a population could jeopardize its long-term survival. Specific studies on harbor porpoises have found higher occurrences of infectious disease in those stranded around European coasts than in less-contaminated Arctic waters (Baker and Martin, 1992; Jauniaux et al. 2002; Jepson et al. 2000; Siebert et al. 2001; Wünschmann et al. 2001). In addition, Beineke et al. (2005) found blubber PCBs (but not DDT) to be significantly correlated with thymic atrophy and splenic depletion in 61 harbor porpoises stranded in the German North and Baltic seas.

Case–control studies are defined as those in which subjects are selected according to their disease status and further classified according to their exposure status (Rothman and Greenland 1998). If the exposure variable is dichotomous, the simple analysis of case–control data yields a cross-product ratio, which is equivalent to the odds of being exposed among the cases divided by the odds of being exposed among the controls [the exposure odds ratio (OR)]. Cases and controls can be randomly selected from a defined source population, provided the sampling is independent of the exposure being studied. Appropriate control selection is important to prevent bias in the OR estimates. Controls should be selected from the same population that gave rise to the cases and independently of their exposure status, because they represent the source population exposure (Smith et al. 1988). For unmatched control selection, the number of exposed and unexposed controls should be in proportion to the amount of exposed and unexposed “animal-time” in the source population. “Animal-time” (analogous to person-time in human studies) represents the average size of the population over the length of the time period being studied. In addition, the time during which a subject is eligible to be a control should be the time in which that individual is also eligible to become a case, if the disease had occurred. Because the controls should represent the source population for the cases, the definition of the source population will assist in identifying the controls. For our study, the source population is defined as the harbor porpoise population in U.K. waters between 1989 and 2002.

Although case–control studies still do not provide causational links between exposure and response, they can provide estimates of average risk (defined as the probability of disease developing in an individual in a specified time period) as estimated by ORs for given exposures > 1. The effect of other variables (confounding factors) on the relationship between exposure and response can be determined using logistic regression. In this study, we used data obtained from the U.K. cetacean strandings scheme [Department of the Environment, Transport and the Regions (DETR) 2000a, 2000b; Jepson et al. 2005]. Harbor porpoises are the most commonly stranded cetacean in U.K. waters (DETR 2000a, 2000b), and previous studies on both subsets of the data from this scheme (Jepson et al. 1999) and the complete data set to date, as used here (Jepson et al. 2005), have found associations between blubber PCB concentrations and death from infectious diseases. Here, we apply the classical case–control approach and use logistic regression to control for confounding factors, to approximate the relative risk of PCB exposure on this population of cetaceans and discuss the validity of using epidemiologic study methods to determine potential population-level effects of contaminant exposure in wildlife.

## Materials and Methods

Details of the harbor porpoise strandings data used in this study have been reported previously (Jepson et al. 2005) and are summarized here. Between July 1989 and December 2002, 1,061 harbor porpoise carcasses stranded in the United Kingdom or caught in commercial fishing nets were necropsied. Detailed pathologic studies were carried out and diagnoses of cause of death determined. Blubber samples were collected from 340 individuals and analyzed for 25 PCB congeners [International Union of Pure and Applied Chemistry (IUPAC) congeners 18, 28, 31, 44, 47, 49, 52, 66, 101, 105, 110, 118, 128, 138, 141, 149, 151, 153, 156, 158, 170, 180, 183, 187, and 194]. The sum of the PCB concentrations (∑25PCB) was converted to a lipid weight basis (milligrams per kilogram lipid) using the proportion of hexane-extractable lipid in the blubber. In order to compare our findings with published studies on blubber PCB concentrations at which adverse effects might be expected (e.g., the LOAEL from Kannan et al. 2000), we also estimated the blubber PCB concentrations based on the commercial formulation Aroclor 1254. We used a conversion factor of 3 × sum of seven congeners (IUPAC congeners 28, 52, 101, 118, 138, 156, and 180) identified by ICES (International Council for the Exploration of the Sea), as described by Jepson et al. (2005). Samples were analyzed at the Centre for Environment, Fisheries and Aquaculture Science Laboratory by gas chromatography with electron-capture detection. The methods incorporate full analytical quality-control protocols involving the analysis of blanks and reference materials within each batch of samples and the preparation of control charts, and have been further validated by participation in the QUASI-MEME (Quality Assurance of Information for Marine Environmental Monitoring in Europe) laboratory proficiency scheme (de Boer and Wells 1997; Wells and de Boer 1994).

Cases were defined as animals that died of an infectious disease (parasitic, bacterial, viral, or mycotic, *n* = 75), and controls as those that died because of acute physical trauma, by-catch, or dystocia (*n* = 161). Because the underlying purpose of controls is to determine the prevalence of past exposure among the catchment population that generated the cases, the controls were a sample from the same population. Table 1 shows the numbers of animals in each of the causes of death categories.

Of the 236 individuals included in the study, 176 were found stranded and 60 were by-catches obtained directly from fishing vessels. The remaining by-caught animals were stranded and subsequently diagnosed as by-catch deaths at postmortem examination. Animals with evidence of acute physical trauma but whose cause of death was clearly infectious disease were included in the cases. Those whose cause of death could not be determined were excluded from the study. Of the 236 individuals examined, 152 were freshly dead and 84 were slightly decomposed. The number of samples stratified by age and sex are shown in Table 2.

Age was determined for 221 individuals by counting growth-layer groups from decalcified tooth sections (Lockyer et al. 2001). For 15 sexually immature animals, age was estimated from body length: < 105 cm, < 1 year; 105–120 cm, 1 year; 121–130 cm, 2 years; > 131 cm, ≥3 years. Energetic status (termed relative body weight) was estimated using the residuals around the best-fit linear regression between ln(body weight) and ln(body length). Animals were also categorized into two seasons based on their date of stranding: October–March and April–September. In addition, six regional areas were identified: southwest England (*n* = 27), east coast of England (*n* = 92), Wales and northwest England (*n* = 73), English Channel (*n* = 5), Scotland (*n* = 36), and Ireland (*n* = 3).

ORs with 95% confidence intervals (CIs) using logistic regression analyses were calculated using R Statistical Package (R Development Core Team 2004) and SPSS version 12.0 (SPSS Inc., Chicago, IL, USA).

## Results

**Figure 1:**
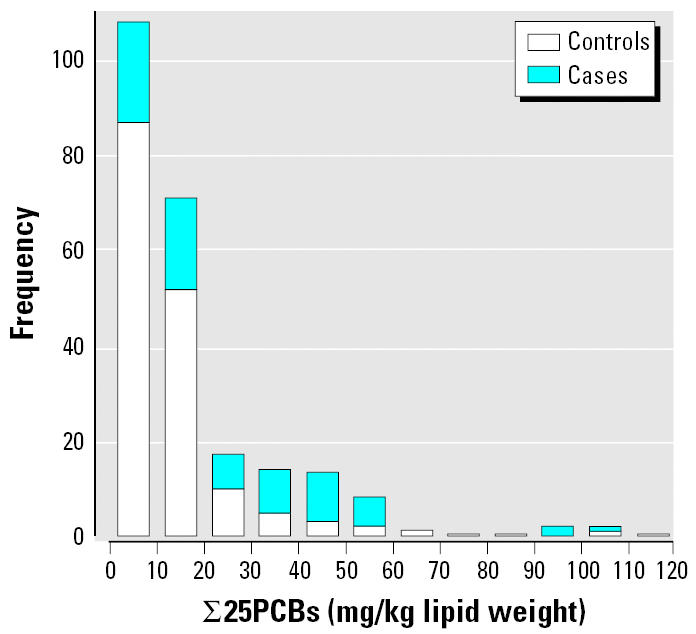
Frequency distribution of ∑25PCBs in the blubber of harbor porpoises selected as cases or controls.

**Figure 2:**
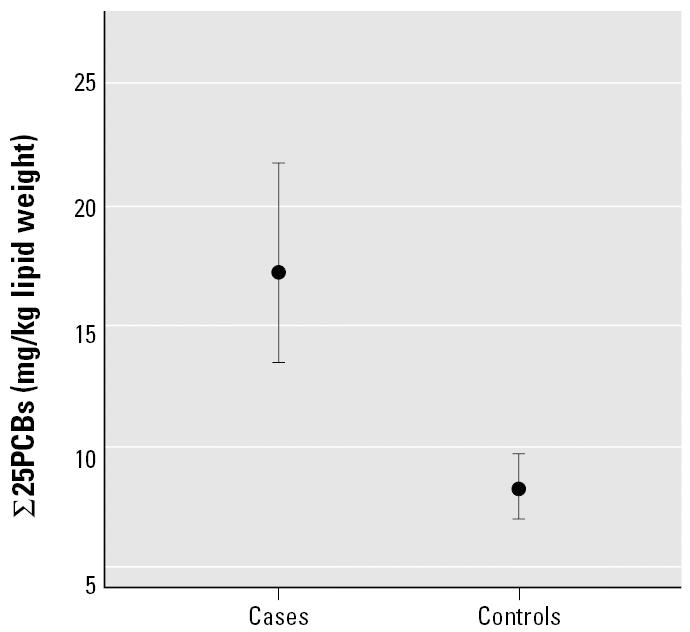
Geometric mean ∑25PCBs (geometric 95% CI) in the blubber of harbor porpoises for cases and controls.

The frequency distributions of the concentrations of ∑25PCB in the blubber of harbor porpoises selected as cases or controls are shown in Figure 1. The group geometric mean ∑25PCB with geometric 95% CIs for the cases and controls are shown in Figure 2. The PCB levels in the cases were significantly higher than in the controls (Welch two-sample *t*-test with unequal variances on log transformed concentrations, *p* < 0.0001).

**Figure 3:**
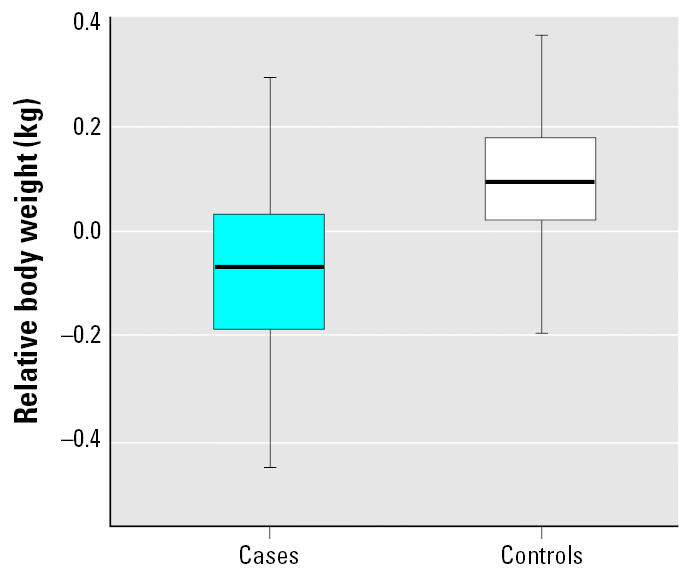
Relative body weight of harbor porpoises [residuals around the best-fit linear regression between ln(body mass) and ln(body length)] among the cases and controls. Values shown are median, 25th–75th percentiles, and minimum–maximum.

Energetic status is an important confounding factor not controlled for in this crude analysis. Relative body weight was significantly lower in the cases than in the controls (Figure 3; Kruskal-Wallis χ^2^ = 52.79, *p* < 0.0001), and this difference must be controlled for in any subsequent analysis. Using a stepwise logistic regression (generalized linear binomial model with a logit link function) to investigate the effect of other factors on cause of death, there was evidence for age, sex, regional, and seasonal confounding (Table 3). Energetic status was the most important factor in predicting the cause of death, with regional and seasonal differences (analysis of deviance, chi-square test for differences between models, terms added sequentially, *p* < 0.05). A stepwise regression using Akiake’s information criterion (AIC) to determine the best model given the data suggested that sex should also be retained in the model. There was no significant interaction between variables. Analysis of the residual deviances suggested that the model was a good fit to the data, and a Hosmer-Lemeshow goodness-of-fit test, in which large values of χ^2^ and small *p*-values indicate a lack of fit of the model, was not significant (χ^2^ = 7.71, *p* = 0.462). After adjusting for the effect of these confounding variables, ∑25PCB remained a highly significant predictor of cause of death.

This logistic regression model (model 1) was then used to estimate the exposure OR and its 95% CI, comparing ∑25PCB in the blubber of the cases and controls and controlling for the effect of energetic status, sex, age, region, and season. The OR was 1.048 (95% CI, 1.02–1.07). These limits do not include the null (1.00) and indicate a slightly higher risk of infectious disease death in harbor porpoises for a 1 mg/kg increase in blubber ∑25PCBs; that is, the odds that an animal will die of infectious disease is 4.8% higher for each unit increase in blubber ∑25PCBs over that of the controls. This is a small unit increase, given the range of exposures and concentrations measured in the porpoises, and may not be the same for a 1–2 mg/kg difference as for a 10–11 mg/kg increase. Table 4 shows the adjusted ORs from this model for differences of blubber ∑25PCBs of between 5 and 60 mg/kg lipid weight, controlling for the confounders. Thus, for a difference of 10 mg/kg, the OR increases to approximately 1.6 (95% CI, 1.56–1.64), which indicates a 60% increase in the risk of infectious disease death.

Because the harbor porpoises that died of infectious disease (cases) had significantly less blubber than those that died of physical trauma (controls), we further investigated the confounding effect of energetic status in our model. Data on total blubber mass (kilograms; from sculp weights) were available for 156 harbor porpoises stranded in the United Kingdom between 1981 and 1987 in addition to sex, total body mass, length, and axillary girth. These four independent variables were then used in a stepwise least-squares linear regression model to determine whether they were good predictors of total blubber mass. The model selection using AIC found that all variables should be retained and the predictive power of the model was good (*R*^2^ = 0.84, *p* < 0.0001). The resulting model equation allowed us to then estimate the total amount of blubber for each animal (where males = 1 and females = 0) as follows:


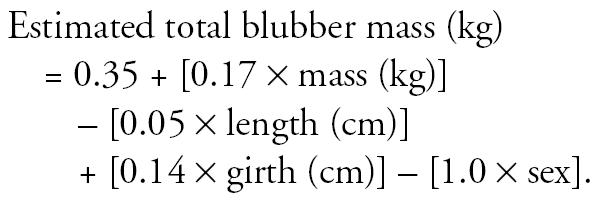


**Figure 4:**
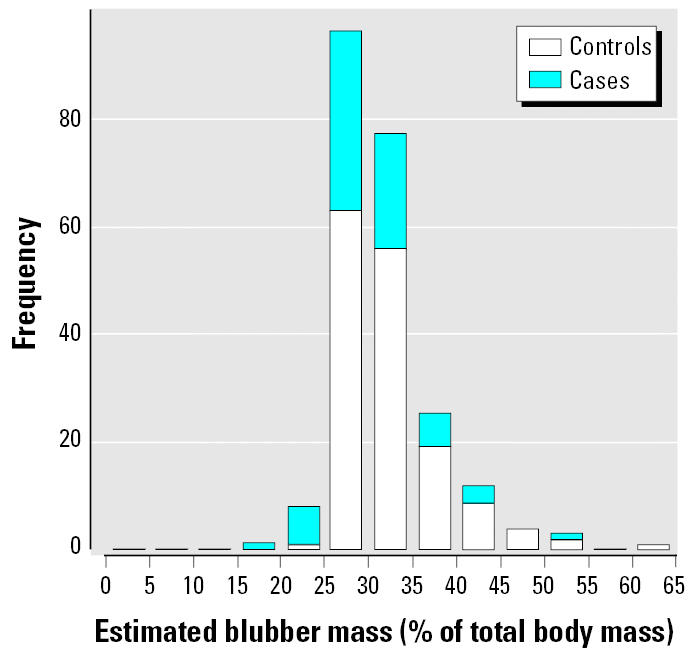
Frequency distribution of estimated blubber mass in harbor porpoises as a percentage of total body mass.

**Figure 5:**
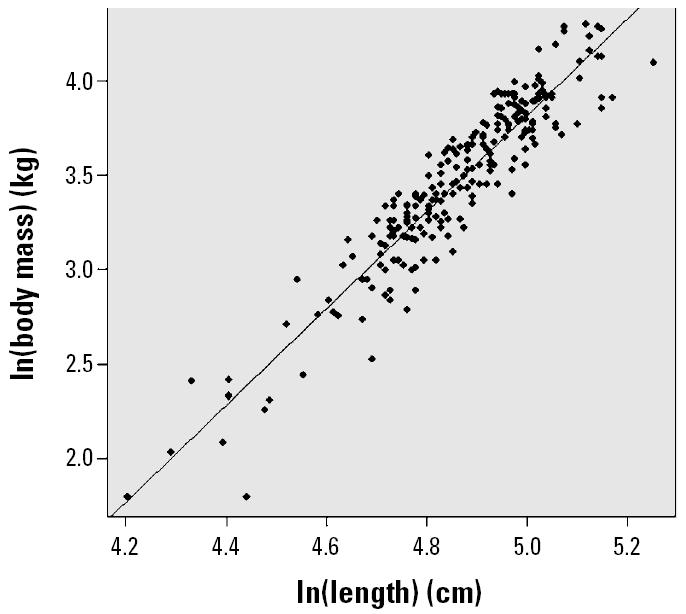
Relationship between ln(body mass) and ln(body length) in harbor porpoises. Line shows best-fit, least-squares linear regression model [ln(mass, kg) = −9.0 + 2.57 × ln(length, cm)].

The frequency distribution of the estimated blubber masses as a percentage of total body mass is shown in Figure 4. These estimates are in line with published data for harbor porpoises where blubber mass was reported to be between 15 and 55% of total body mass (Koopman et al. 2002; McLellan et al. 2002). The best-fit least-squares regression equation from the relationship between ln(mass) and ln(length), as shown in Figure 5, was then used to determine a predicted total body mass for each individual. This standardized the study animals to a population mean for a given length. Thus, assuming that the decrease in total body mass in nutritionally stressed animals was a consequence of blubber depletion (Koopman et al. 2002) and that any contaminants in the blubber are then concentrated in the remaining fat layer, we can estimate what the concentration of PCBs in the blubber would have been if the animals had been in optimal body condition for their size. This was carried out by calculating a “standardized” blubber mass for each individual as follows:


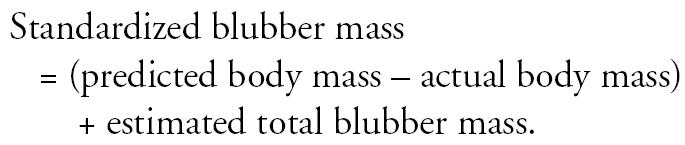


Finally a standardized concentration of ∑25PCB (mg/kg lipid) was estimated as


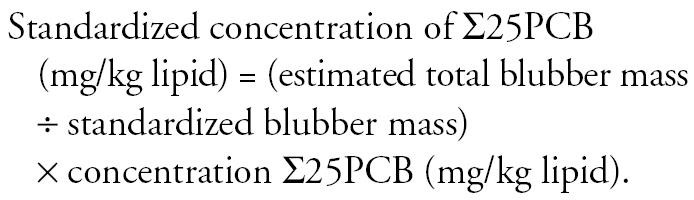


Table 5 shows the results from the second logistic regression model, again with cause of death as the dependent variable, but this time including the standardized ∑25PCB concentration as an independent variable, in addition to the other confounder factors (sex, region, and season). ∑25PCBs remained a significant factor in predicting cause of death (*p* = 0.025), although the level of significance was lower, after accounting for the effect of the other potential confounders. In addition, sex was now significant (*p* = 0.036) in this model. The resulting OR was lower than in the first model at 1.02 (95% CI, 1.00–1.03). Again, the CI did not include the null but suggested a slightly lower risk of infectious disease death (2% for a 1 mg/kg increase in blubber ∑25PCBs). Table 6 shows the adjusted ORs for differences of blubber ∑25PCBs between 5 and 60 mg/kg lipid using this model (model 2). For a difference of 10 mg/kg, the OR is now 1.18 (an 18% increase in risk; 95% CI, 1.16–1.2). A 50% increase in risk does not occur until differences in blubber ∑25PCBs reach approximately 25 mg/kg lipid, and a doubling of risk occurs at approximately 45 mg/kg lipid.

In order to compare our results with the published LOAELs for marine mammals, we repeated the model 2 analysis, which gave us the most conservative OR estimates, using the Aroclor 1254 equivalents as the concentration of PCBs in blubber. On this basis, a 50% increase in risk occurs at around 45 mg/kg, and a 2-fold increase at around 80 mg/kg.

## Discussion

This is the first study to quantify the risk of infectious disease in relation to exposure to PCBs in a marine mammal using a classical case–control approach. This epidemiologic approach estimates the average risk of the outcome of interest (infectious disease mortality) at the population level. Our aim was to determine whether observations from one population indicated whether, on average, the risk of infection was higher with increasing PCB exposure, and indeed our results do support this hypothesis. It should be noted that the resulting risk estimates may not be applicable to all individuals within the population or, indeed, to different pathogen exposures.

If harbor porpoises that die of infectious disease are more likely to be washed ashore and reported than are those that die from other causes, this would produce bias in the OR estimates. Unfortunately, this is a difficult phenomenon to test empirically, and we can only assume that reporting rates are not related to cause of death. In addition, if the controls included animals whose cause of death was related to exposure (i.e., animals with high PCBs are more likely to die of physical trauma), then this would bias the OR downward. However, the bias could also be in the opposite direction. If the control group underestimates the exposure in the source population, then this will overestimate the OR. Perhaps animals that died of physical trauma were less likely to be exposed to PCBs. Avoiding such selection bias continues to be a challenge for epidemiologists (Flanders and Austin 1986; Goldstein et al. 1989; Lin and Paik 2001; Smith et al. 1988; Sutton-Tyrrell 1991), and although we have endeavored to choose controls independently of their exposure status, we cannot rule out the effects of such selection biases on our results.

There are also some pitfalls in equating ORs to relative risks. The risk (or probability) of an event (or disease) occurring in a specified period of time is very difficult to measure in many situations, particularly for wildlife, whereas the odds of an event (calculated as the number of events divided by the number of nonevents), as demonstrated in this study, are quite tangible. Although ORs do not approximate well to the relative risk when the initial risk (i.e., the prevalence of the outcome of interest) is high (Davies et al. 1998), when the initial risk is lower (as in this study), odds and risks are very similar (see Deeks 1996).

In addition, other environmental contaminants are correlated with concentrations of PCBs in the blubber of harbor porpoises (Kuiken et al. 1994). These include DDT and its metabolites, organochlorine pesticides, and mercury. These contaminants were also measured in the blubber of the harbor porpoises included in this study (Jepson 2003). On average, ∑25PCBs made up 62% of the total contaminant concentrations in the blubber, with DDT accounting only for a further 18%. In addition, hepatic total butyltins and mercury concentrations were measured, but these were not related to cause of death (Jepson 2003). If other contaminants are accounting for the relationships observed, then these would have to be highly correlated with blubber ∑25PCBs. PCBs have been demonstrated in a wide variety of species to be the most immunotoxic of the POPs measured, exerting effects through AhR-dependent and AhR-independent pathways (Silkworth et al. 1984; Smithwick 2003). Because PCBs dominate the blubber contaminant profiles and recent studies have shown that PCBs alone are immunotoxic to harbor seals (Hammond et al. 2005), we have inferred that PCB exposure is the most probable candidate for increasing the risk of infection in this species. However, it is quite plausible that effects are in fact due to the mixture of contaminants to which these animals are exposed and not a consequence of exposure to PCBs alone.

A problem to overcome in this analysis was the potential confounding effect of energetic status in the cases. We took two approaches to investigate this. The first was to include energetic status and other potential confounding variables as covariates in the logistic regression model. Because PCB concentrations are known to be associated with blubber thickness in marine mammals (Aguilar and Borrell 1994a) and the cases and controls as sampled had different distributions of energetic status (used as a surrogate for blubber mass), including this in the model as a covariate allowed the sample means for each group to be adjusted to determine whether the findings were solely attributable to the fact that the cases in the sample had a lower energetic status than did the controls. After controlling for this difference, the cause of death was still significantly predicted by blubber PCB concentrations. In addition to this approach, we also adjusted the blubber PCB concentrations to a “standardized” concentration based on predicting what each animals’ blubber level would have been if it had been in optimal condition. This method indicated that blubber PCBs remained significant predictors of cause of death but resulted in lower ORs to estimate the risk of infectious disease death after PCB exposure. Clearly, there are considerable areas of uncertainty in using this second approach because the added error from estimating standardized concentrations was not accounted for. In addition, the elevation in contaminant concentrations is perhaps not as high as a purely concentrative model would suggest (Aguilar et al. 1999). However, by using both approaches, we would still infer that the risk of infection is more likely to be due to PCB exposure than because emaciated animals had higher blubber PCB levels.

We have shown that the risk of death from infectious disease in harbor porpoises is associated with increasing PCB exposure, but further research is necessary to ascertain whether this relationship is causal. Most of the PCBs stored in the blubber of marine mammals are a legacy from the mother transferred during lactation (Hickie et al. 1999) rather than direct uptake from prey. Using the conservative estimates from our models, the increased risk of infectious disease death is high (> 2.0) when differences of ∑25PCB exceed 45 mg/kg lipid weight. The LOAEL in marine mammals calculated by Kannan et al. (2000), based on diverse primary data sources, was 17 mg/kg lipid based on total PCBs as the sum of 42 congeners (Boon et al. 1987). Although the results of our study are therefore not directly comparable with this value (representing a guide level rather than any absolute threshold) because the total PCBs were estimated using slightly different methods, it provides an indication of an approximate threshold level for effects determined by others. Again, using the conservative OR estimates, our study found that a 2-fold increase risk of infectious disease death does not occur until concentrations exceed 80 mg/kg lipid Aroclor 1254 equivalent. These levels are also higher than the above threshold value because the toxicity end points measured here are the most extreme, that is, mortality from infection. Kannan et al. (2000) were estimating effect levels for responses at the lower end of the physiologic range, such as disruption of vitamin A and thyroid hormone concentrations, suppression of natural killer cell activity, and reduced proliferative responses of lymphocytes to mitogens. These end points are at the sublethal end of the range of toxic effects and will impair the health of the animal, causing morbidity. Although we do not have good dose–response data for immune function effects in marine mammals, the published data do indicate that effects are more severe as exposures increase (Hammond et al. 2005; Lahvis et al. 1995). Thus, at the highest exposure levels, effects on both innate and acquired immunity and, indeed, on cell-mediated and humoral functions could result in more severe immunosuppression and premature mortality from infection after pathogen exposure.

Beineke et al. (2004) optimized lymphocyte-transformation assays and investigated cytokines using polymerase chain reaction in harbor porpoises, but suppression of these responses after PCB exposure has not yet been reported. Extrapolating from effects on other marine mammal species and the assumption that the increased risk of infectious disease in relation to blubber PCB levels is mediated through effects on immunity may be flawed. Indeed, seal species appear to vary considerably in their susceptibility to effects of PCBs on innate immunity: gray seals (*Halichoerus grypus*) appear to be much more resistant to exposure than harbor seals (Hammond et al. 2005). Using our ORs to assess the risk of infectious disease after exposure to PCBs in other marine mammal species would also be inappropriate. Until more detailed data on immune function effects of PCBs in harbor porpoises are available, our results should be interpreted in this light. With this caveat in mind, we can investigate which other populations of harbor porpoises outside the North Sea are likely to be at increased risk. Examples from published studies that report comparable data include animals incidentally caught in Scandinavian (Danish and Norwegian) waters between 1987 and 1991, in which total blubber PCBs ranged from 4 to 65 mg/kg lipid weight, with a median of 20 mg/kg (Kleivane et al. 1995). Three animals from the Baltic Sea sampled between 1989 and 1990 had levels of 26–47 mg/kg lipid (Falandysz et al. 1994). In the Black Sea, blubber PCB levels in 10 harbor porpoises sampled in 1993 ranged from 3 to 39 mg/kg lipid weight (Tanabe et al. 1997). PCBs in the blubber of 45 harbor porpoises from the coasts of Washington, Oregon, and California in the United States in the mid-1980s ranged from 2 to 129 mg/kg lipid weight (Calambokidis et al. 1984). Jarman et al. (1996) found levels of PCBs in harbor porpoises from British Columbia (*n* = 6, 1987–1989) of 5–17 mg/kg lipid weight, whereas animals from the California coast (*n* = 3) had levels of 4.5–42 mg/kg lipid weight. All of these populations are therefore likely to include some individuals with blubber levels sufficiently high (> 25 mg/kg lipid) to have an increased risk of infection. Although most of these studies relate to the late 1980s, we found no significant decline in PCBs in North Sea harbor porpoises since this time (Jepson 2003). In contrast, harbor porpoises from the Greenland population [total blubber PCBs < 5 mg/kg lipid; *n* = 177; 1986–1988 (Borrell et al. 1999, 2004; Granby and Kinze 1991)], the Canadian northeast Pacific [< 10 mg/kg lipid; *n* = 6; 1987 (Aguilar et al. 2002)], and those around the Faroe Islands (total blubber PCBs 8–13 mg/kg lipid; *n* = 6; 1987–1988 (Borrell 1993)] do not have a significantly increased risk.

In an ecologic context, it is often impractical to determine the incidence of infection in an unexposed population. If the background incidence rate is negligible, then a doubling of the risk is still negligible and thus unlikely to be important at the population level. Although the background incidence rate is not quantifiable for harbor porpoises, a precautionary approach would warn that high exposure levels might cause significant excess mortality. Although the production and use of PCBs in Europe have been banned since the 1980s (Smith and Gangolli 2002), substantial amounts of PCBs are still in use, largely because of the long service life of PCB-containing equipment such as large transformers and capacitors. There are also considerable quantities of PCBs in storage awaiting disposal. Thus, the exposure of long-lived marine mammals to these chemicals worldwide is likely to continue. Our results could also be used in further risk assessment models for population-level effects where dose–response data are difficult to obtain for free-living marine mammals such as the harbor porpoise.

The approach we have taken has not been widely used in ecotoxicologic studies, despite its potential power and relevance. We have demonstrated the general applicability of the case–control approach for studies using marine mammal strandings data. Only such long-term schemes can provide suitable data, and this study illustrates the need for their continuation into the future.

## Figures and Tables

**Table 1:** Number of harbor porpoises by cause of death category among the cases and controls.

Cause of death	No.
Cases	
Pneumonia, parasitic	20
Pneumonia, bacterial	2
Pneumonia, fungal	3
Pneumonia, mixed pathogens	18
Pneumonia, unknown cause	2
Generalized bacterial infection	20
Gastritis/enteritis	4
Generalized viral infection	1
Meningo-encephalitis	2
Other infection (e.g., myositis, otitis media)	3
Controls	
By-catch	126
Physical trauma	29
Dystocia	6

**Table 2:** Total number of harbor porpoise cases and controls stratified by sex and age class.

	Adults		Immatures		Unknown		
	Male	Female	Male	Female	Male	Female	Total
Cases (infectious disease deaths)	17	19	17	22	0	0	75
Controls (physical trauma deaths)	38	22	59	41	0	1	161
Total	55	41	76	63	0	15	236

**Table 3:** Analysis of deviance table from the relationship between cause of death (infectious disease and physical trauma) and potential confounding factors using stepwise logistic regression (binomial model with a logit link function).

Potential confounding factor	df	Deviance	Residual df	Residual deviance	*p*-Value
Energetic status	1	52.65	229	237.1	< 0.0001
Sex	1	2.75	228	234.3	0.097
Region	5	16.26	223	218.1	0.006
Season	1	9.04	222	209.0	0.003
∑ 25PCB	1	16.32	221	192.7	< 0.0001

df, degrees of freedom. Terms were added sequentially, first to last.

**Table 4:** Adjusted ORs for risk of infectious disease death in harbor porpoises for differences of standardized blubber ∑25PCB between 5 and 60 mg/kg lipid weight from model 1.

Difference in ∑ 25PCB	OR (∑PCB_1_ − ∑ PCB_0_) a	95% CI
5	1.265	1.234–1.297
10	1.601	1.561–1.641
15	2.025	1.976–2.076
20	2.563	2.500–2.627
25	3.242	3.163–3.324
30	4.102	4.001–4.205
35	5.190	5.063–5.321
40	6.567	6.405–6.732
45	8.308	8.104–8.517
50	10.512	10.254–10.777
55	13.300	12.973–13.635
60	16.827	16.414–17.251

a Adjusted for energetic status, sex, region, and season. ∑PCB_1_, exposed; ∑ PCB_0_, unexposed.

**Table 5:** Analysis of deviance from the relationship between cause of death (infectious disease and physical trauma) and potential confounding factors including standardized ∑25PCB (mg/kg lipid weight) as a dependent variable (binomial model with a logit link function).

Potential confounding factor	df	Deviance	Residual df	Residual deviance	*p*-Value
Sex	1	4.40	222	275.4	0.036
Region	5	20.86	217	254.5	0.001
Season	1	7.88	216	246.7	0.005
Standardized ∑ 25PCB	1	4.99	215	214.7	0.025

df, degrees of freedom. Terms were added sequentially, first to last.

**Table 6:** Adjusted ORs for risk of infectious disease death in harbor porpoises for differences of standardized blubber ∑25PCB between 5 and 60 mg/kg lipid weight from model 2.

Difference in ∑ 25PCB	OR (∑PCB_1_ − ∑ PCB_0_)^a^	95% CI
5	1.087	1.071–1.103
10	1.181	1.164–1.198
15	1.283	1.264–1.302
20	1.394	1.374–1.415
25	1.515	1.493–1.538
30	1.646	1.622–1.671
35	1.789	1.763–1.816
40	1.944	1.916–1.973
45	2.113	2.082–2.144
50	2.296	2.262–2.330
55	2.495	2.458–2.532
60	2.711	2.671–2.751

a Adjusted for sex, region, and season. ∑PCB_1_, exposed; ∑PCB_0_, unexposed.
